# Antibacterial Activity and Mechanism of *Taxillμs chinensis* (DC.) Danser and Its Active Ingredients

**DOI:** 10.3390/ijms251910246

**Published:** 2024-09-24

**Authors:** Yanjing Feng, Silu Huang, Shengying Zhu, Bo Gao

**Affiliations:** 1 School of Life Sciences, Jilin University, Changchun 130012, China; fengyj23@mails.jlu.edu.cn (Y.F.); jiayiy24@mails.jlu.edu.cn (S.H.); zhusy22@mails.jlu.edu.cn (S.Z.); 2 Key Laboratory for Molecular Enzymology and Engineering of Ministry of Education, Jilin University, Changchun 130012, China

**Keywords:** *Taxillμs chinensis* (DC.) Danser, 4-indolecarbaldehyde, antimicrobial activity, antibacterial mechanism

## Abstract

*Taxillμs chinensis* (DC.) Danser is a traditional Chinese herbal medicine. It has not been reported regarding antibacterial active ingredients and mechanisms of action. However, the Chinese patent medicine Yinhua Miyanling Tablets containing *Taxillμs chinensis* has an obvious anti-infective effect in our patent. Therefore, we speculate that *Taxillμs chinensis* may have antibacterial activity. The purpose of this paper is to study the antibacterial effect and mechanism of *Taxillμs chinensis* and find active compounds with antibacterial activity and a mechanism. We studied the antibacterial effect and mechanism of *Taxillμs chinensis* extract. The compounds in the ethyl acetate extract of *Taxillμs chinensis* were preliminarily identified by UPLC-Q-Orbitrap and analyzed by mass spectrometry. Above all, the antibacterial effect and antibacterial mechanism of the active components of *Taxillμs chinensis* were determined. Finally, we found, for the first time, that *Taxillμs chinensis* has a good antibacterial effect and ethyl acetate extract has the best effect. In addition, we found, for the first time, that it has an active component, 4-indolecarbaldehyde, and the component has a good broad-spectrum antibacterial effect. Above all, the active chemical 4-indolecarbaldehyde of *Taxillμs chinensis* can destroy the bacterial structure, make it unable to maintain normal morphology, and significantly increase the number of deaths. In short, *Taxillμs chinensis* has an antibacterial effect, and one of its main antibacterial components is 4-indolecarbaldehyde. The antibacterial mechanism of *Taxillμs chinensis* and 4-indolecarbaldehyde is related to the change in bacterial membrane permeability.

## 1. Introduction

In recent years, the incidence of infectious diseases caused by bacteria and fungi has increased year by year, and most of them are treated with antibiotics [1]. However, with the wide application of antibiotics, clinical treatment is also facing many difficulties. Due to the lax control of antibiotics, the irrational use of antibiotics and synthetic antibiotics in the social population has caused some pathogenic bacteria to mutate and become drug-resistant strains [2], or because of the long-term use of antibiotics, the sensitive bacteria are inhibited, and the insensitive bacteria (such as fungi, etc.) take the opportunity to reproduce and grow in the body, resulting in a double infection [3]. Antimicrobial resistance poses a serious public health threat to the progress of infectious diseases, cancer treatment, organ transplantation, and intensive care [4]. The World Health Organization (WHO) report pointed out that antimicrobial resistance poses a major threat to human health around the world [5]. Globally, drug-resistant infections caused by antimicrobial resistance cause about 700,000 deaths each year. If effective intervention is not taken, it is expected to cause 10 million deaths by 2050 and global economic losses of up to 100 trillion USD [6]. Searching for antibacterial molecules with high efficiency and low toxicity from natural plants is a research hotspot in natural product chemistry. Natural drugs generally have multiple antibacterial targets, and the possibility of drug resistance is small [7,8,9]. A variety of different components contained in natural medicines can also produce other unexpected therapeutic effects with their unique synergistic effects [10]. Some studies have also shown that some natural drugs have the effect of reversing the drug resistance of drug-resistant strains [11]. In recent years, more and more reports have been reported that the mechanism of some natural drugs to exert bacteriostatic action is related to the improvement of body immunity [12]. Therefore, with the unique advantages of natural drugs in the field of bacteriostasis, obtaining high-efficiency and low-toxicity antibacterial agents and conducting in-depth research on their activity and mechanism of action can provide new ways and new ideas for the clinical treatment of infectious diseases.

Yinhua Miyanling Tablets are mainly composed of *Lonicera japonica* Thunb., *Scutellaria barbata* D. Don, *Polygonum aviculare* L., *Dianthus superbus* L., *Pyrrosia lingua* (Thunb.) Farwell., *Clematis armandii* Franch., *Plantago asiatica* L., *Lophatherum gracile* Brongn., *Taxillμs chinensis* (DC.) Danser, and *Juncus effusus* L. It has the effect of removing dampness, clearing heat, and detoxifying and adding the function of detoxification and tonic [13]. Modern pharmacological studies have confirmed that Yinhua Miyanling Tablets have antibacterial, anti-inflammatory, diuretic, and other effects and are used for the treatment of diseases such as urinary tract infections [14,15,16,17,18,19,20]. Compared to modern antibiotics, the traditional antibacterial Chinese patent medicine Yinhua Miyanling Tablets has more advantages in long-term use and does not easily produce bacterial resistance. Wang Xinlei et al. confirmed that Yinhua Miyanling Tablets had obvious antibacterial endotoxin activity in vivo or in vitro [18]. Li Yun et al. reported that Yinhua Miyanling Tablets have a good antibacterial effect on *Staphylococcus* and are not affected by the existing clinical resistance [19]. However, at present, in Yinhua Miyanling Tablets, many components are reported for antibacterial effect [21,22,23,24,25,26], but *Taxillμs chinensis* has not been reported.

*Taxillμs chinensis* (DC.) Danser, is a plant of Taxillus Van Tiegh of Loranthaceae of Santalales [27]. Because of its rich flavonoids, alkaloids, terpenoids, polysaccharides, organic acids, and other functional substances, it has become one of the important medicinal plants in traditional Chinese medicine. In the traditional clinical application, *Taxillμs chinensis* has the functions of tonifying the liver and kidney, dispelling rheumatism, strengthening bones and muscles, nourishing blood, preventing miscarriage, and lowering blood pressure. At present, the related research on the pharmacology of *Taxillμs chinensis* shows that it also has good effects on the treatment of hypertension, cancer, stroke, inflammation, hyperlipidemia, diabetes, and neuroprotection and has high clinical value [28].

In this paper, *Taxillμs chinensis* was selected as the main research object. Five kinds of organic solvents were used to extract, and the solvent extracts with the strongest antibacterial activity were selected. The chemical composition of the extract of *Taxillμs chinensis* was analyzed by ultra-high-performance liquid chromatography-quadrupole-orbitrap mass spectrometry (UPLC-Q-Orbitrap). Highly sensitive detection and chemical structure identification, in-depth elucidation of the chemical composition and the discovery of monomeric compounds with antibacterial activity, and exploring the antibacterial mechanism of its monomeric compound are accomplished for *Taxillμs chinensis* clinical application of anti-infection to provide a scientific basis.

## 2. Results

### 2.1. Antimicrobial Effect of Different Extracts of Taxillμs chinensis

The antimicrobial effect of different extracts of *Taxillμs chinensis* is shown in Table 1. It can be seen from Table 1 that different extracts showed a certain degree of antimicrobial activity against the tested strains at the preset concentration. The antimicrobial strength of the extract was as follows: ethyl acetate extract > acetone extract > n-butanol extract > chloroform extract > petroleum ether extract. Among them, the ethyl acetate extract had the best antimicrobial and bactericidal activity. The minimum inhibitory concentration (MIC) value of *Bacillus spizizenii*, *Staphylococcus aureus* (*S. aureus*), and *Aspergillus niger* was 2.5 mg/mL, and the minimum bactericidal concentration (MBC) value was 5 mg/mL, showing good antimicrobial and bactericidal activity. The antimicrobial and bactericidal activities against *Escherichia coli* (*E. coil*) and *Penicillium italicum* were slightly weaker. The MIC and MBC values were 5 mg/mL and 10 mg/mL, respectively. Subsequent studies were extracted with ethyl acetate.

### 2.2. The Antibacterial Mechanism of Ethyl Acetate Extract of Taxillμs chinensis

#### 2.2.1. Analysis of the Growth Curve

**Figure 1:**
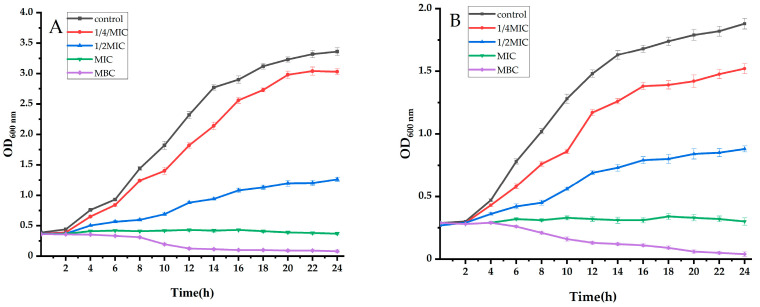
Effects of ethyl acetate extract of *Taxillμs chinensis* on the growth curves of *S. aureus* (**A**) and *E. coil* (**B**) (*p* < 0.05, compared to the control group).

In this experiment, the growth curves of *S. aureus* and *E. coil* were determined by turbidimetry, and the absorbance of the bacterial suspension was determined by a microplate reader. The concentration of the bacterial suspension was inferred by the absorbance of the bacterial suspension. The measured absorbance value and its corresponding culture time are plotted in Figure 1. *S. aureus* and *E. coil* were used as the test bacteria, and 0.1% dimethyl sulfoxide (DMSO) was used as a control group to study the growth of pathogenic bacteria under different concentrations of ethyl acetate extract of *Taxillμs chinensis* and to further verify the antibacterial activity of ethyl acetate extract of *Taxillμs chinensis*. The results are shown in Figure 1A. In the blank control of *S. aureus*, a standard S-shaped curve of bacterial growth was shown. When 1/4XMIC ethyl acetate extract was added to the Luria–Bertani (LB) medium, the lag phase was prolonged compared to the control group, but the overall growth pattern remained typical. However, when the concentration of 1/2XMIC of the ethyl acetate extract of *Taxillμs chinensis* was added, the growth of the bacteria was significantly inhibited, showing a longer lag period, and the final amount of bacteria was only 37.5% of the control. When the concentration of the MIC was added, the OD_600nm_ value was unchanged, the cell growth was completely inhibited, and there was no sign of proliferation. In addition, when the concentration of the MBC was added, the OD_600nm_ value gradually decreased, and the cells died.

Similarly, for *E. coil* (Figure 1B), the bacteria of the control group showed a clear S-type. After the addition of 1/4XMIC drug, the logarithmic phase of the bacteria was delayed, but it still showed a typical growth pattern. After adding 1/2XMIC drug, the amount of bacteria increased slightly, and the final amount of bacteria was 46.8% of the control group. After adding MIC and MBC drugs, the growth of *E. coil* was completely inhibited. The results showed that the low concentration of ethyl acetate extract of *Taxillμs chinensis* could delay the growth of bacteria. With the increase in concentration, the growth of bacteria was significantly inhibited, indicating that the ethyl acetate extract of *Taxillμs chinensis* could inhibit the growth and reproduction of pathogenic bacteria, showing a strong antibacterial effect.

#### 2.2.2. Analysis of Extracellular Conductivity

**Figure 2:**
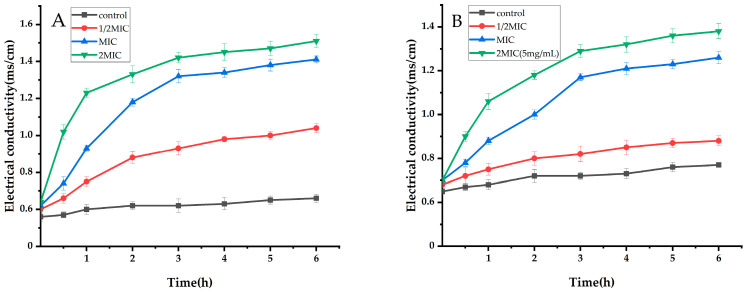
Effect of ethyl acetate extract of *Taxillμs chinensis* on the extracellular conductivity of *S. aureus* (**A**) and *E. coil* (**B**) (*p* < 0.05 (unless the 1/2XMIC group of group (**B**) compared to the control group).

When the bacterial membrane is damaged, the selective permeability changes, and a large number of intracellular ions flow out, so that the extracellular conductivity increases. Therefore, the change in extracellular conductivity can be measured to reflect whether the cell membrane is damaged [29]. Using *S. aureus* and *E. coil* as test bacteria, the effect of the ethyl acetate extract of *Taxillμs chinensis* on the conductivity of bacterial suspension was studied, as shown in Figure 2. For *S. aureus* (Figure 2A), there was no significant change in the conductivity of the bacterial suspension in the control group at different treatment times. However, after the addition of MIC and 2XMIC ethyl acetate extracts, the conductivity of the bacterial suspension increased significantly and was always higher than that of the control group (*p* < 0.05), and before 3 h, the increasing trend was significant. However, after 3 h, the increase trend tended to be gentle. The reason was that the drug inhibited the proliferation of bacteria, the number of bacterial colonies no longer increased, and the bacteria were still lysing, so the conductivity change value became smaller. When the concentration was 1/2XMIC, the conductivity of the bacteria was significantly lower than that of the control group (*p* < 0.05), which was significantly lower than that of MIC and 2XMIC, indicating that the greater the concentration of the drug treatment, the greater the change in conductivity, the more extracellular ions, and the more serious the bacterial membrane damage.

For *E. coil* (Figure 2B), after adding MIC and 2XMIC drugs, the conductivity of the bacteria increased significantly, and it was always higher than that of the control group (*p* < 0.05). When the concentration was 1/2XMIC, there was no significant change in the conductivity of the cells compared to the control group (*p* > 0.05), indicating that, when the concentration of the ethyl acetate extract of *Taxillμs chinensis* was 1/2XMIC, there was no significant effect on the permeability of the bacteria.

In summary, the results showed that the extracellular conductivity of the two bacteria increased significantly after treatment with the ethyl acetate extract of *Taxillμs chinensis*, but the Δσ of *S. aureus* was greater than that of *E. coil*, and more extracellular ions were discharged. It is speculated that the ethyl acetate extract of *Taxillμs chinensis* does more serious damage to the cell membrane of *S. aureus* and a better antibacterial effect.

#### 2.2.3. Analysis of Extracellular Potassium Ions (K^+^)

**Figure 3:**
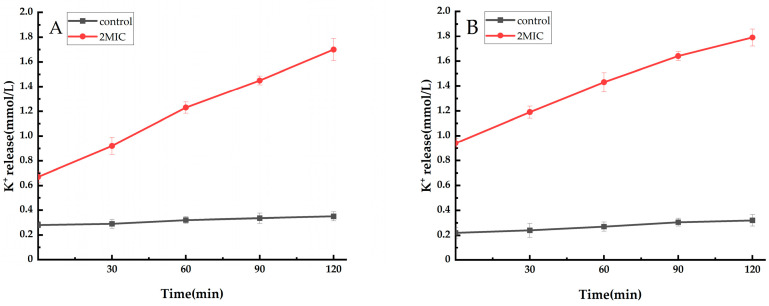
Effect of ethyl acetate extract of *Taxillμs chinensis* on extracellular potassium ions of *S. aureus* (**A**) and *E. coil* (**B**) (*p* < 0.05, compared to the control group).

Potassium homeostasis is essential for bacterial survival and plays an important role in maintaining the balance of osmotic pressure on both sides of the cell membrane and maintaining normal life activities. The cell membrane is an important barrier to maintain potassium homeostasis. If the cell membrane is damaged, a large amount of intracellular potassium ions will flow out. Therefore, it is possible to determine whether the cell membrane is damaged by measuring changes in extracellular potassium ions [30]. Using *S. aureus* and *E. coil* as test bacteria, the effect of the ethyl acetate extract of *Taxillμs chinensis* on the extracellular potassium ion concentration is shown in Figure 3. After *S. aureus* and *E. coil* were treated with 2XMIC *Taxillμs chinensis* ethyl acetate extract, the intracellular potassium ions were released to the extracellular, and the potassium ion content in the bacterial suspension was significantly increased, and both were higher than the control group (*p* < 0.05), which was positively correlated with time, while the content of the extracellular potassium ions in the control group remained stable and the detection amount was extremely low. In the 0–120 min period, the extracellular K^+^ change of *S. aureus* was 1.03, and the extracellular K^+^ change of *E. coil* was 0.85, indicating that, during this period, *S. aureus* compared to *E. coil*, the K^+^ that flowed from the intracellular to the extracellular was more, and the damage of the cell membrane was greater. In short, the ethyl acetate extract of *Taxillμs chinensis* can change the permeability of the cell membrane of *S. aureus* and *E. coil*, resulting in a large number of small molecules leaking to the extracellular, and the effect on *S. aureus* is better.

### 2.3. UPLC-Q-Orbitrap Was Used to Detect the Active Components of Ethyl Acetate Extract of Taxillμs chinensis

**Figure 4:**
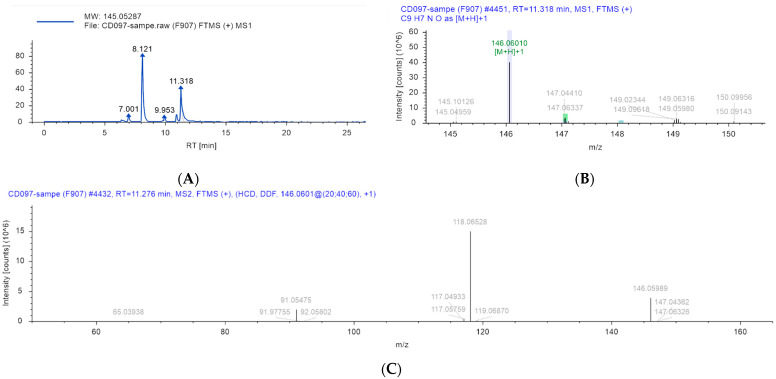
Identification of the structure of 4-indolecarbaldehyde: (**A**) liquid chromatography, (**B**) the first-order mass spectrometry, and (**C**) the second-order mass spectrometry.

UPLC-Q-Orbitrap was used to detect the main chemical components of the ethyl acetate extract with a better antibacterial effect. The chromatographic and mass spectrometry information of the main chemical components of the ethyl acetate extract with a better antibacterial effect was obtained. The collected multi-level mass spectrometry information was used to preliminarily identify 102 compounds in the ethyl acetate extract of *Taxillμs chinensis* by sweeping the library score, as shown in Supplementary Table S1. By comparing with the standard, the chemical composition of 4-indolecarbaldehyde (RT = 11.32 min) was accurately identified, as shown in Table 2 and Figure 4. 4-Indolecarbaldehyde is an important pharmaceutical and organic chemical intermediate, which can synthesize many compounds with physiological or pharmacological activities. Studies have shown that 100 μM 4-indolecarbaldehyde is non-toxic to HepG2 cells and 3T3-L1 Preadipocytes. It has shown that 4-indolecarbaldehyde has low cell toxicity and certain human safety [31,32]. Studies have shown that the three indole alkaloids isolated and identified from *Pseudomonas aeruginosa* have strong antibacterial effects [33]. However, there is no report on the antibacterial activity of 4-indolecarbaldehyde. Therefore, we continue to explore the antibacterial effect and mechanism of 4-indolecarbaldehyde.

### 2.4. Antimicrobial Effect of Active Compound 4-Indolecarbaldehyde in Taxillμs chinensis

#### 2.4.1. Antibacterial Effect

4-Indolecarbaldehyde is shown in Table 3. It can be seen from Table 3 that 4-indolecarbaldehyde showed a certain degree of antimicrobial activity against 12 tested strains. Among the seven fungi tested, 4-indolecarbaldehyde also had a certain inhibitory effect. Among the above five bacteria, the antibacterial effect on Gram-positive bacteria (*Bacillus spizizenii*, *S. aureus*, and Methicillin-resistant *Staphylococcus aureus* (MRSA)) was better, and the half-maximal inhibitory concentration (IC_50_) value of MRSA was the smallest. In summary, 4-indolecarbaldehyde showed a good broad-spectrum antimicrobial effect. According to Figure 2 and Figure 3, the ethyl acetate extract of *Taxillμs chinensis* can inhibit the activity of *S. aureus* and *E. coil*, and the effect on *S. aureus* is better. Therefore, we used the “super bacteria” MRSA with strong drug resistance as the test bacteria to explore the antibacterial mechanism of the compound 4-indolecarbaldehyde.

#### 2.4.2. Effect of MRSA Cell Membrane Permeability

**Figure 5:**
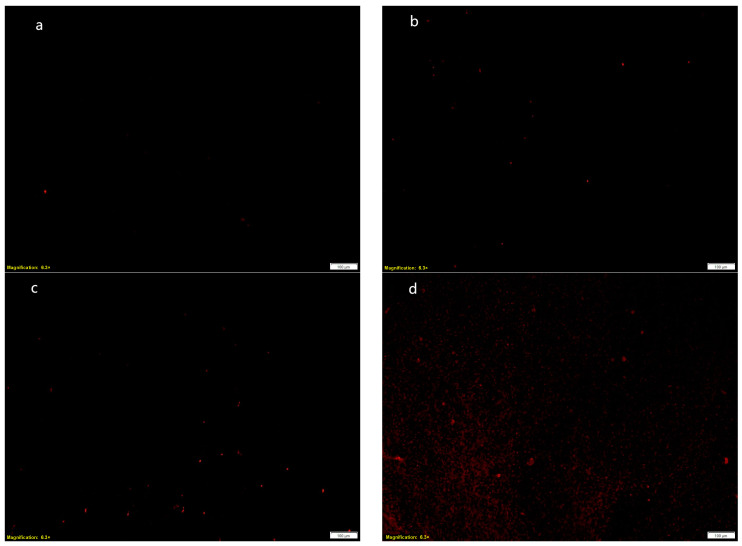
Inverted fluorescence microscopy of MRSA treated with 4-indolecarbaldehyde: (**a**) The control group treated with 0.1% DMSO. (**b**) The 1/2XMIC treatment group. (**c**) The MIC treatment group. (**d**) The MBC treatment group (magnification: 6.3×).

After MRSA was treated with 1/2XMIC, MIC, and MBC, the propidium iodide (PI) staining results are shown in Figure 5. For normal MRSA bacteria, PI could not pass through the bacterial membrane, could not bind to the bacterial DNA, and was excited by fluorescence. However, for the bacteria with damaged bacterial membranes, the membrane permeability changed. PI could pass through the bacterial membrane and bind to the bacterial DNA, and then, fluorescence could be observed. Therefore, the fluorescence intensity observed by PI staining could be used to reflect the damage to the bacterial membrane [34].

Figure 5a was the control group treated with 0.1% DMSO. From Figure 5, it can be seen that the bacteria in the control group did not observe fluorescence, indicating that the MRSA biofilm was not destroyed and could block the PI dye into the cell. When MRSA was treated with 4-indolecarbaldehyde (Figure 5b) at a concentration of 1/2XMIC, a small amount of fluorescence could be observed. It was speculated that the bacteria were in a bad environment, which affected the normal growth of the bacteria and caused a small amount of death of the bacteria. When MRSA was treated with 4-indolecarbaldehyde (Figure 5c) at a concentration of MIC, the fluorescence intensity was significantly higher than that of the blank control group, indicating that, after the drug treatment, the cell membrane permeability changed, PI entered the cell, and combined with DNA, and was excited by fluorescence. When MRSA was treated with 4-indolecarbaldehyde (Figure 5d) at the concentration of MBC, a large area of fluorescence could be observed, indicating that a large number of bacterial membranes were damaged, and the bacteria died at the MBC concentration. The above phenomena indicated that 4-indolecarbaldehyde may affect the normal growth and metabolism of bacteria by changing the cell membrane permeability of MRSA, and with the increase in the drug concentration, the fluorescence intensity increased, and the number of bacteria damaged by the bacterial membrane increased.

#### 2.4.3. The Effect of MRSA Mortality

**Figure 6:**
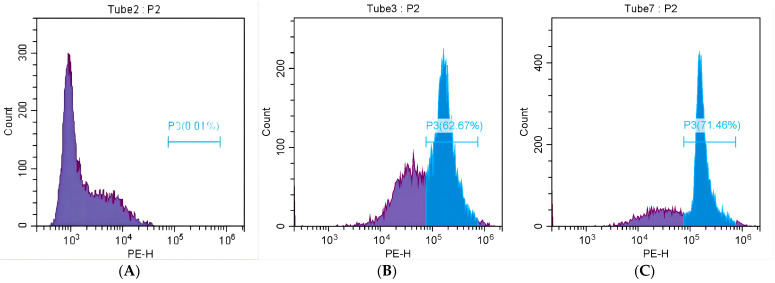
Inverted fluorescence microscopy of MRSA treated with 4-indolecarbaldehyde: (**A**) The control group treated with 0.1% DMSO; (**B**) the 3XMIC treatment group; (**C**) the MBC treatment group (The P3 (blue) region represents dead cells).

Flow cytometry was used to analyze the changes in the growth and reproduction stages of bacterial cells. PI is a nucleic acid dye that cannot penetrate the intact cell membrane but can penetrate the damaged cell membrane of late apoptotic cells and dead cells, causing the nucleus to dye red [35]. The results of MRSA lethality detected by flow cytometry are shown in Figure 6. It can be observed that the fluorescence images of the MRSA blank group (Figure 6A) were all located on the left side, and the fluorescence intensity was weak. The P3 region was only 0.01%, indicating that the bacteria grew normally, and the bacterial membrane was not damaged. After MRSA (Figure 6B) was treated with 4-indolecarbaldehyde at 3XMIC, the fluorescence map moved to the right, and the fluorescence intensity increased significantly. The P3 region was 62.67%, indicating that more than half of the MRSA biofilm was damaged at this time. The permeability of the biofilm changed, and PI passed through the damaged biofilm and bound to DNA; after the treatment of MRSA (Figure 6C) with 4-indolecarbaldehyde in MBC, the P3 region was 71.46%, indicating that, when the concentration of 4-indolecarbaldehyde increased, the fluorescence intensity increased, and the number of bacterial membrane damage increased. More PI passed through the damaged bacterial membrane and bound to DNA, causing the number of bacterial deaths to increase. The above phenomena indicated that 4-indolecarbaldehyde MRSA can affect the normal growth and metabolism of bacteria by changing the permeability of the bacterial membrane, thus exerting a bacteriostatic effect.

## 3. Materials and Methods

### 3.1. Plants and Strains 

*Taxillμs chinensis* (N 20°09′~25°31′, E 109°45′~117°20′) was purchased from Jilin Yilutang Trading Co., Ltd (Jilin, China). *Escherichia coli* (*E. coil*) (American Type Culture Collection (ATCC) 25922), *Staphylococcus aureus* (*S. aureus*) (ATCC25923), *Bacillus spizizenii* (ATCC6633), Methicillin-resistant *Staphylococcus aureus* (MRSA) (ATCC43300), *Aspergillus niger* (ATCC16888), *Mucor racemosus* (ATCC22365), *Penicillium italicum* (ATCC48955), *Rhizopus oryzae* (ATCC96382), *Geotrichum candidum* (ATCC90686), *Aspergillus flavus* (ATCC26214), *Botrytis cinerea* (GDMCC 3.47) and *Rhizopus* sp. (GDMCC 3.230) all were purchased from the Guangdong Microbial Culture Collection Center (GDMCC), (Guangzhou, China). 

### 3.2. Activation and Culture of Strains

Activation and culture of bacteria: The strains of *S. aureus*, *E. coil*, and *Bacillus spizizenii* preserved in the laboratory in a −80 °C refrigerator were taken out (MRSA freeze-dried powder was taken out and dissolved in a small amount of Brain Heart Infusion broth (BHI broth)). In a sterile environment, the strains in the cryopreservation tube were picked with the inoculation ring and inoculated into the nutrient agar plate (MRSA inoculated into the BHI agar plate). The strains were cultured in a 37 °C incubator for 18–24 h. One to three single colonies were picked with a sterile inoculation ring and transferred to a nutrient broth (NB) culture and a 37 °C water bath shaker bed overnight culture [36,37].

Activation and culture of fungi: The *Aspergillus niger* and *Penicillium* strains preserved in the laboratory in a −80 °C refrigerator were taken out, and the strains in the frozen storage tube were selected with an inoculation ring in a sterile environment and inoculated into glucose potato agar and cultured in a constant temperature incubator at 28 °C for 2–3 days. One to three single colonies were picked with a sterile inoculation ring, transferred to glucose potato water, and cultured in a 28 °C water bath shaker for 2 days.

### 3.3. Preparation of Ethanol Extract of Taxillμs chinensis and Different Extracts from the Ethanol Extract of Taxillμs chinensis

The powder of *Taxillμs chinensis* (over 60 mesh sieve) was added with 95% ethanol (solid–liquid ratio 1:20) and ultrasonically extracted at 60 °C for 60 min. After the extraction, the filtrate was collected by vacuum filtration and concentrated at 45 °C in a rotary evaporator to obtain the crude ethanol extract of *Taxillμs chinensis*. At room temperature, the crude ethanol extract of *Taxillμs chinensis* was transferred to the liquid separation funnel with a small amount of distilled water. First, the crude ethanol extract of *Taxillμs chinensis* was extracted three times with petroleum ether. After the petroleum ether extract was combined, the petroleum ether extract was concentrated by a rotary evaporator. Subsequently, it was extracted with chloroform, ethyl acetate, n-butanol, and acetone in turn. The petroleum ether, chloroform, ethyl acetate, n-butanol, and acetone extraction components were dried to a constant weight at 45 °C in a vacuum drying oven and stored at −20 °C for the detection of their antibacterial activity.

### 3.4. Determination of MIC and MBC of Different Extracts of Taxillμs chinensis

#### 3.4.1. Determination of MIC

MIC was determined by the broth dilution method but slightly modified [38,39]. The strains were taken out from the refrigerator at −80 °C, restored to room temperature, activated on the slant medium, and incubated at 37 °C for 24 h. One or two typical colonies were selected from the inclined surface of the activated bacteria and inoculated into a 20 mL Mueller–Hinton (MH) broth medium. The culture was shaken at 37 °C for 4 h, and the bacterial concentration was determined and adjusted to 10^5^ CUF/mL [40]. The extract was dissolved in DMSO and adjusted to the appropriate concentration. The sterile 96-well culture plate was taken out, and 100 μL sterile MH broth medium was added to each well, and 100 μL liquid medicine was added to the first hole of each row. After mixing, 100 μL mixed liquid was added to the second hole, and 10 holes were added according to the double dilution method. Another hole plus levofloxacin was taken as a positive control [41], and 1 hole of 0.1% DMSO was used as a negative control, and 100 μL bacterial liquid was added to each hole. Take 0.5% 2,3,5-triphenyte-trazoliumchloride (TTC) solution, add 5 μL TTC solution to each well, mix the hole, and culture at 37 °C. Three groups of parallel tests were set up according to the above sampling method.

#### 3.4.2. Determination of MBC

A total of 90 μL of bacterial suspension was added to 96-well plates, and then, 10 μL of sample solution with MIC and the above concentration gradient was added to each well in turn and mixed (10 μL of sample solution was added, and the final concentrations obtained in the experiment were MIC, 2XMIC, 3XMIC, 4XMIC, and 5XMIC). After 10 h of culture at 37 °C, 10 μL of sample solution was taken out from each well and evenly coated in an agar culture dish at 37 °C, and after 18 h of culture, the growth of the strain was observed, and the minimum drug concentration when the colony ≤ 5 was the MBC value. Parallel operations were run 3 times, and the results were the average [42].

### 3.5. Antibacterial Effect of Ethyl Acetate Extract of Taxillμs chinensis

#### 3.5.1. Determination of the Growth Curve

The bacterial growth curve was measured by a microplate reader (Epoch, BioTek Instruments, Inc., Winooski, VT, USA) and colony-forming unit assay but slightly modified [43]. The bacterial suspension of 3.2-activated *S. aureus* and *E. coli* was prepared, and the concentration was adjusted to 10^7^ CFU/mL. Then, MH liquid medium with different concentrations of ethyl acetate extract of *Taxillμs chinensis* was added to make the final concentrations of 1/4XMIC, 1/2XMIC, MIC, and MBC, respectively, and 0.1% DMSO was used as the control, shaken well, and cultured at 37 °C. The optical density of OD_600nm_ was measured every 2 h to monitor cell growth [44].

#### 3.5.2. Determination of Extracellular Conductivity 

*S. aureus* and *E. coli* cultured to the logarithmic phase were centrifuged at 5000× *g* for 6 min, washed 3 times with 5% glucose, and resuspended until the conductivity of the bacterial suspension was close to 5% glucose. The concentration of the bacteria was adjusted to 10^8^ CFU/mL, and then, different concentrations of ethyl acetate extract of *Taxillμs chinensis* were added to make the final concentrations of 1/2XMIC, MIC 2XMIC, and 0.1% DMSO as the control. The conductivity of 0 to 6 h was measured by a conductivity meter (DDSJ-318, Shanghai Meiyingpu Instrument Manufacturing Co., Ltd., Shanghai, China) [45].

#### 3.5.3. Determination of the Potassium Ion Content in Bacterial Suspension

First, 20 mL of bacterial solution cultured to the logarithmic growth phase were taken, centrifuged at 3000 rpm for 10 min, and then, the bacteria were collected. After washing the collected bacteria with sterile phosphate-buffered saline (PBS), the bacteria were resuspended in 10 mL PBS buffer again, and the bacterial concentration was adjusted to 10^8^ CFU/mL. The control group was a PBS bacterial suspension without the ethyl acetate extract of *Taxillμs chinensis,* and the experimental group was a PBS bacterial suspension with the final concentration of 2XMIC of the ethyl acetate extract of *Taxillμs chinensis*. The control group and the experimental group were simultaneously placed at 37 °C, 180 rpm in a constant temperature oscillation box. Samples were taken every 30 min, centrifuged for 10 min at 4500 rpm, and the supernatant was taken. The content of potassium ions in the bacterial suspension was detected by a microplate reader according to the operation method of the potassium ion kit, and the content of potassium ions in the bacterial suspension was measured three times in parallel [46].

### 3.6. UPLC-Q-Orbitrap Was Used to Study the Active Components of the Ethyl Acetate Extract of Taxillμs chinensis

To a precisely weighed 200 mg of the ethyl acetate extract of *Taxillμs chinensis*, 1 mL of methanol:water (8:2, *v*/*v*) was added, vortexed, and mixed. Two zirconia grinding beads were added, ground, and extracted for 3 min and centrifuged at 4 °C for 10 min with a centrifugal force of 20,000× *g*; the supernatant was filtered with a 0.22 um filter membrane, and the filtrate was taken for analysis. The mass spectrometry detection conditions were as follows: using an electrospray ionization source (ESI), positive and negative ion switching scanning, scanning range of 150.0~2000.0 *m*/*z*, 3.8 kV (positive) electrospray voltage, capillary temperature of 300 °C, nitrogen temperature of 350 °C, and data acquisition 30 min. The chromatographic detection conditions were as follows: RP-C18 chromatographic column (150 × 2.1 mm, 1.8 μm), flow rate of 0.30 mL/min, 0.1% formic acid aqueous solution as the aqueous phase, 0.1% formic acid acetonitrile as the organic phase, methanol as the needle washing solution, column oven temperature of 35 °C, automatic sampler temperature of 10.0 °C, injection needle height of 2.00 mm, automatic sampler injection volume of 5.00 μL, the gradient used in the separation was 0 min: aqueous phase/organic phase = 98/2 (*v*/*v*); 5 min: water/organic phase = 4/1 (*v*/*v*); 10 min: water phase/organic phase = 1/1 (*v*/*v*); 15 min: water phase/organic phase = 1/4 (*v*/*v*); 20 min: water phase/organic phase = 5/95 (*v*/*v*); 26 min: aqueous phase/organic phase = 98/2 (*v*/*v*); and data acquisition 30 min [47,48]. Mass spectrometer (Q Exactive high-resolution mass spectrometer, Thermo Fisher Scientific, Waltham, MA, USA) and a chromatograph (UltiMate 3000 RS, Thermo Fisher Scientific, Waltham, MA, USA) were used.

### 3.7. Antibacterial Effect of Active Compounds in Taxillμs chinensis

#### 3.7.1. Determination of MIC, MBC, and IC_50_ of 4-Indolecarbaldehyde

Determination of MIC: Refer to step in Section 3.4.1.

Determination of MBC: Refer to step in Section 3.4.2.

Determination of IC_50_: Firstly, the activated MRSA strains were inoculated into the sterile MH broth medium and cultured in a 37 °C water bath for 12 h. Then, 180 μL of the bacterial solution was added to the sterile 96-well culture plate, and finally, 20 μL of different concentrations of the drug solution was added. At the same time, the 4-indolecarbaldehyde administration group, blank control, and negative control were set up 3 times in parallel. The 96-well plates were incubated at 37 °C for 12 h, and the absorbance was measured by a microplate reader at 595 nm. The inhibition rate (%) was calculated according to the formula [49]:

$$IR\;(\%)\;=\;((I_{\mathrm{sc}}\;-\;I_{c})\;-\;(I_{\mathrm{lm}}\;-\;I_{c}))/(I_{\mathrm{nc}}\;-\;I_{c})\;\times \;100\%$$

I_sc_ = solvent control hole OD value; I_c_ = the corresponding supernatant OD value;

I_lm_ = liquid medicine hole OD value; I_nc_ = negative control OD value.

The sample concentration and inhibition rate were plotted, and the linear regression equation was obtained.

#### 3.7.2. Detection of MRSA Cell Membrane Permeability

The concentration of MRSA suspension was adjusted to 10^7^ CFU/mL, and then, MH liquid medium with different concentrations of 4-indolecarbaldehyde was added to make the final concentrations of MIC and MBC, respectively, and 0.1% DMSO was used as a control, shaken, and treated at 37 °C for 4 h. Then, 1 mL bacterial solution was taken, washed with double-distilled water 3 times, centrifuged, and the supernatant was removed. Next, 500 μL PI/RNase staining solution was added and stained in the dark for 15 min. After centrifugation, the PI/RNase staining solution was removed, washed twice with sterile double-distilled water, resuspended with 500 μL double-distilled water, and sliced. The fluorescence inverted microscope (LW300LFT, Shanghai Cewei optoelectronic Technology Co., Ltd., Shanghai, China) was used to observe and take photos (PI/RNase fluorescent fuel was easy to quench, and the exposure time should not be too long) [50].

#### 3.7.3. The Mortality of MRSA Was Detected by Flow Cytometry

The concentration of the MRSA suspension was adjusted to 10^7^ CFU/mL, and then, different concentrations of 4-indolecarbaldehyde MH liquid medium were added to the final concentration of 3XMIC and MBC, and 0.1% DMSO as the control was shaken and treated at 37 °C for 4 h. Then, 1 mL of bacterial solution was resuspended in PBS 3 times, centrifuged, and then washed twice with sterile double-distilled water and centrifuged. The cells were resuspended with precooled 70% ethanol to make the cells completely disperse and fixed overnight at 4 °C. Next, 500 μL of bacterial solution was centrifuged, washed twice with double-distilled water, centrifuged to remove the supernatant, and 500 μL of PI/RNase dye solution was added and incubated in the dark for 15 min (dyeing time should not be too long). It was washed twice with double-distilled water, the PI/RNase dye solution was removed, it was resuspended with 500 μL double-distilled water, and finally detected by flow cytometry (CyoFLEXS, Beckman Coulter, Inc., Brea, CA, USA) [51].

### 3.8. Statistical Analysis

Each test was carried out in triplicate. The results are expressed as the mean ± SD. The significant difference was analyzed at the level of *p* < 0.05 via one-way ANOVA by SPSS (26.0) software.

## 4. Conclusions

This paper mainly explored the antibacterial active components and antibacterial mechanism of the natural plant *Taxillμs chinensis*, including the comparison of antibacterial effects of *Taxillμs chinensis* species extracts, the antibacterial effect and antibacterial mechanism of ethyl acetate extracts, the analysis and identification of active components of ethyl acetate extracts of *Taxillμs chinensis*, and the antibacterial effect and antibacterial mechanism of monomer compounds. Firstly, we found that the five extracts of *Taxillμs chinensis* had certain inhibitory effects on bacteria and fungi, and the ethyl acetate extract had the best inhibitory effect. In the study of the antibacterial effect and mechanism of the ethyl acetate extract of *Taxillμs chinensis*, when the ethyl acetate extract of *Taxillμs chinensis* acted on the bacteria, a large number of small molecular substances was leaked, indicating that the ethyl acetate extract of *Taxillμs chinensis* could change the permeability of the cell membrane, and the effect on *Staphylococcus aureus* was better. In addition, we used UPLC-Q-Orbitrap to preliminarily identify 102 compounds in the ethyl acetate extract of *Taxillμs chinensis* and performed mass spectrometry analysis to obtain the effective compound 4-indolecarbaldehyde. In the study of the antibacterial effect and mechanism of 4-indolecarbaldehyde, 4-indolecarbaldehyde showed a good broad-spectrum antibacterial effect, and the antibacterial mechanism of 4-indolecarbaldehyde was related to the change in bacterial membrane permeability. The above studies have proven that *Taxillμs chinensis* has the potential to become an effective drug in bacteriostasis, and we have deeply explored the antibacterial ability of the 4-indolecarbaldehyde and its effect on the permeability of the bacterial membrane, providing a scientific basis for the clinical application of the anti-infection of *Taxillμs chinensis*.

## 5. Patents

The 4-Indolecarbaldehyde compound with antibacterial activity in *Taxillμs chinensis*, preparation method, and its application. Bo Gao, Silu Huang, Zi’an Qiao, Changmin Liu, and Min Yan. ZL 2020 1 1218127.2.

## Figures and Tables

**Table 1:** MIC and MBC of different extracts of *Taxillμs chinensis* on 5 kinds of strains.

Microbe Species	Concentration (mg/mL)									
	Ethyl Acetate Extract		Acetone Extract		n-Butanol Extract		Chloroform Extract		Petroleum Ether Extract	
	MIC	MBC	MIC	MBC	MIC	MBC	MIC	MBC	MIC	MBC
*Staphylococcus aureus*	2.5	5	2.5	10	5	10	10	10	>20	-
*Bacillus spizizenii*	2.5	5	2.5	10	5	10	10	20	>20	-
*Escherichia coli*	5	10	5	10	5	10	10	20	>20	-
*Aspergillus niger*	2.5	5	5	5	5	10	10	10	>20	-
*Penicillium italicum*	5	10	5	10	5	10	10	20	>20	-

**Table 2:** 4-Indolecarbaldehyde structure information (RT [min] is the chromatographic retention time, Molecular Weight is the accurate molecular weight identified by the database, and mzCloud Best Match is the mzCloud database matching score (the higher the value, the higher the credibility of the identified results)).

Structure	Name	Formula	RT [min]	Molecular Weight	mzCloud Best Match
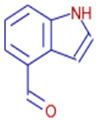	4-Indolecarbaldehyde	C_9_H_7_NO	11.83	145.0528	86.0

**Table 3:** Antimicrobial effect of 4-indolecarbaldehyde on 12 kinds of bacteria.

Microbe Species	Concentration (μg/mL)		
	4-Indolecarbaldehyde		
	MIC	MBC	IC_50_
*Escherichia coli*	256	1024	147.22
*Bacillus spizizenii*	128	512	72.74
*Staphylococcus aureus*	128	512	69.35
MRSA	128	512	67.07
*Aspergillus niger*	256	1024	165.35
*Aspergillus flavus*	256	1024	173.26
*Penicillium*	256	1024	183.53
*Botrytis cinerea*	256	512	144.68
*Rhizopus* sp.	256	512	153.49
*Rhizopus oryzae*	128	1024	76.23
*Geotrichum candidum*	128	512	64.49
*Mucor racemosus*	64	512	40.25

## Data Availability

Data are contained within the article and the Supplementary Materials.

## Supplementary Materials

The following supporting information can be downloaded at https://www.mdpi.com/article/10.3390/ijms251910246/s1.
