# Supplementary material for: Antibacterial Activity and Mechanism of Taxillμs chinensis (DC.) Danser and Its Active Ingredients
Source: Int J Mol Sci. 2024 Sep 24;25(19):10246. doi: 10.3390/ijms251910246 (PMC11477399; doi:10.3390/ijms251910246)

# Total ion chromatograms of natural products in CD097 samples

Note : Column 1 black is the positive and negative total ion flow diagram superposition diagram, column 2 red is the positive ion mode total ion flow diagram, and column 3 green is the negative ion mode total ion flow diagram.

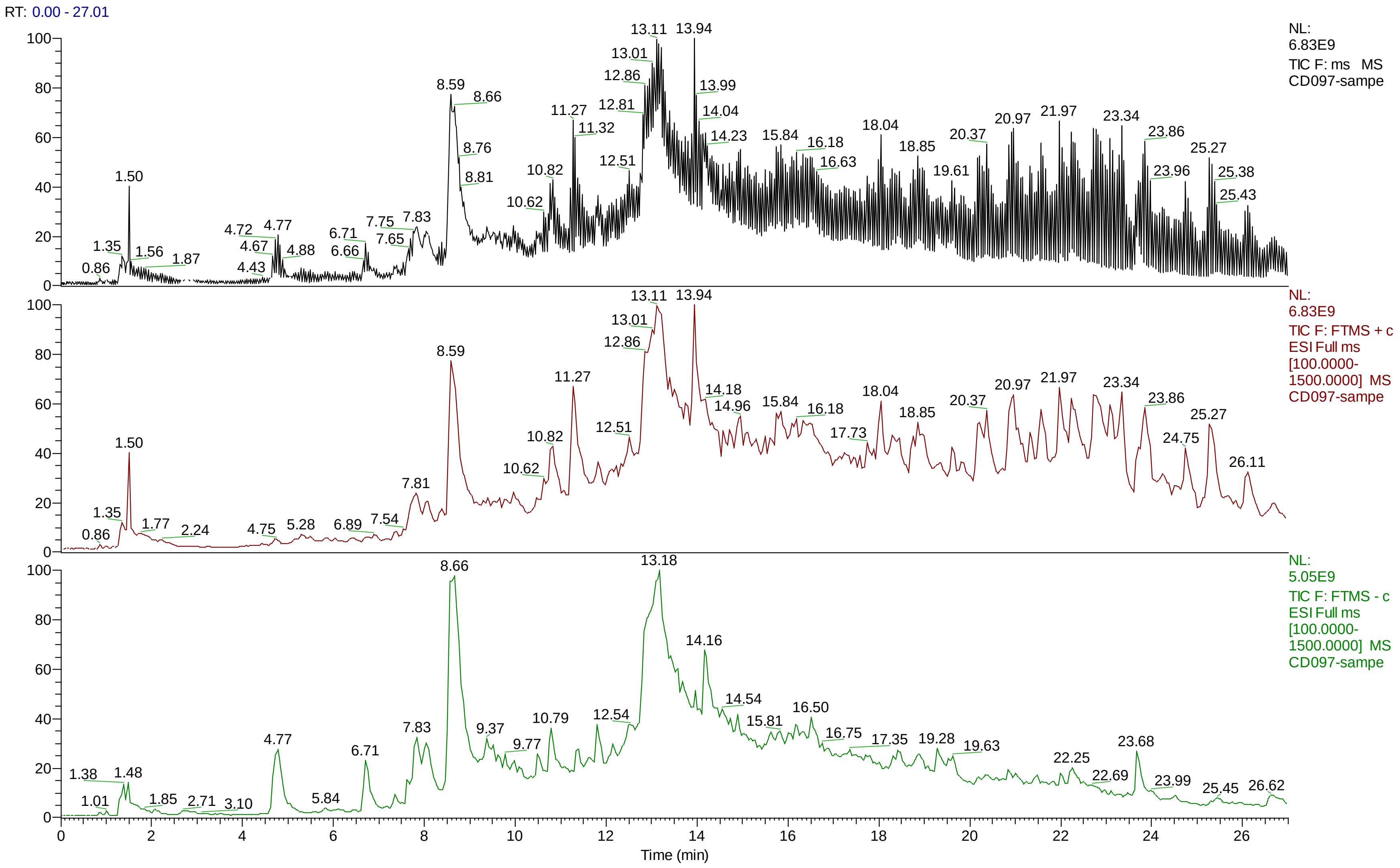

Supplement: Supplementary file 1 [file ijms-25-10246-s001.zip › Supplementary Figure S1Total ion chromatograms of natural products in CD097 samples.pdf]
