# Supplementary material for: Antibacterial Activity and Mechanism of Taxillμs chinensis (DC.) Danser and Its Active Ingredients
Source: Int J Mol Sci. 2024 Sep 24;25(19):10246. doi: 10.3390/ijms251910246 (PMC11477399; doi:10.3390/ijms251910246)
Supplement: Supplementary file 1 [file ijms-25-10246-s001.zip › Supplementary Table S1.pdf]

Supplementary Table S1. Chromatographic, mass spectrometry information and structural inference of the main chemical components of ethyl acetate extract of *Taxillμs chinensis*

NO.	RT [min]	Calc (m/z)	Found (m/z)	diff(ppm)	Formula	Fragment Ions	Ion Mode	Compounds
1	1.187	129.10145	129.10164	1.47171	$C_4H_{11}N_5$	88，71，60	Pos	Metformin
2	1.302	145.08513	145.08508	-0.34463	$C_5H_{11}N_3O_2$	128，111，104，87，86，60	Pos	4-Guanidinobutyric acid
3	1.481	196.0583	196.05762	-3.46836	$C_6H_{12}O_7$	159，129，105，99，75，59	Neg	Gluconic acid
4	1.661	192.06339	192.06282	-2.96777	$C_7H_{12}O_6$	173，155，127，99，85，71，57	Neg	D-(-)-Quinic acid
5	2.067	123.03203	123.03228	2.03199	$C_6H_5NO_2$	106，96，80	Pos	Nicotinic acid
6	2.818	122.04801	122.04827	2.13031	$C_6H_6N_2O$	106，96，80	Pos	Nicotinamide
7	2.919	145.07389	145.07387	-0.13786	$C_6H_{11}NO_3$	126，15，102，100，83，77，58	Neg	4-Acetamidobutanoic acid
8	3.148	112.02728	112.02737	0.80338	$C_4H_4N_2O_2$	96，70	Pos	Uracil
9	4.112	145.07389	145.07408	1.30968	$C_6H_{11}NO_3$	104，86	Pos	4-Acetamidobutanoic acid
10	6.452	219.11067	219.11061	-0.27383	$C_9H_{17}NO_5$	146，88，71	Pos	Pantothenic acid
11	6.604	187.06333	187.06359	1.38990	$C_{11}H_9NO_2$	146，118，91	Pos	Indole-3-acrylic acid
12	6.715	154.02661	154.02647	-0.90893	$C_7H_6O_4$	125，109，91，81，65	Neg	2,3-Dihydroxybenzoic acid
13	6.758	306.07395	306.0742	0.81680	$C_{15}H_{14}O_7$	247，181，16，139，111，65	Neg	(-)-Epigallocatechin
14	6.784	173.99868	173.99885	0.97702	$C_6H_6O_4S$	154，137，109，93，79，59	Neg	4-Phenolsulfonic acid
15	7.042	191.06948	191.06963	0.78505	$C_9H_9N_3O_2$	160，132 ，114，96	Pos	Carbendazim
16	7.043	154.02661	154.02667	0.38954	$C_7H_6O_4$	123，109，91，81，65	Neg	2,3-Dihydroxybenzoic acid
17	7.105	142.02661	142.02683	1.54901	$C_6H_6O_4$	125，115，97，83，69	Pos	cis,cis-Muconic acid
18	7.295	112.05243	112.05288	4.01598	$C_6H_8O_2$	95，85，71，67，57	Pos	Sorbic acid
19	7.363	152.04734	152.04747	0.85500	$C_8H_8O_3$	135，107，95	Pos	3-Methylsalicylic acid
20	7.496	146.05791	146.05783	-0.54773	$C_6H_{10}O_4$	101，83	Neg	2-Methylglutaric acid
21	7.625	159.08954	159.08944	-0.62858	$C_7H_{13}NO_3$	116，102，58	Neg	N-Acetylvaline
22	7.717	211.11095	211.11086	-0.42632	$C_{13}H_{13}N_3$	195，119，94，91，77	Pos	N,N'-Diphenylguanidine
23	7.747	113.08406	113.08441	3.09504	$C_6H_{11}NO$	96，72，69	Pos	Caprolactam
24	7.826	306.07395	306.07384	-0.35939	$C_{15}H_{14}O_7$	271，231，163，139，135，123，111，93，	Pos	(-)-Epigallocatechin
25	8.212	146.05791	146.05778	-0.89006	$C_6H_{10}O_4$	127，101，81	Neg	2-Methylglutaric acid
26	8.409	340.07943	340.07949	0.17643	$C_{15}H_{16}O_9$	117，133，105，81	Neg	Esculin
27	8.617	290.07904	290.0786	-1.51683	$C_{15}H_{14}O_6$	245，203，179，151，	Pos	Catechin

						123, 95, 69		
28	8.707	154.02661	154.02547	-7.40132	$C_7H_6O_4$	109, 108, 91, 69	Neg	Gentisic acid
29	8.713	176.06847	176.06763	-4.77087	$C_7H_{12}O_5$	157, 129, 115, 85	Neg	2-Isopropylmalic acid
30	8.869	179.09463	179.09459	-0.22335	$C_{10}H_{13}NO_2$	180, 138, 121, 103, 93, 92, 77	Pos	N-Acetyltyramine
31	9.358	160.07356	160.0736	0.24989	$C_7H_{12}O_4$	141, 115, 97, 59	Neg	2,2-Dimethylglutaric acid
32	9.428	178.02661	178.02669	0.44937	$C_9H_6O_4$	161, 151, 135, 133, 105, 91, 77	Pos	Esculetin
33	9.444	354.09508	354.09494	-0.39537	$C_{16}H_{18}O_9$	233, 191, 93, 71	Pos	Chlorogenic acid
34	9.668	180.04226	180.04162	-3.55472	$C_9H_8O_4$	151, 135, 91, 69, 59	Neg	Caffeic acid
35	9.927	270.05282	270.05278	-0.14812	$C_{15}H_{10}O_5$	243, 215, 149, 121, 91, 68	Pos	Genistein
36	9.956	290.07904	290.07849	-1.89603	$C_{15}H_{14}O_6$	245, 203, 179, 151, 123, 95, 69	Pos	Catechin
37	10.201	133.05276	133.05297	1.57832	C_8H_7NO	106, 93, 79	Pos	2-Oxindole
38	10.335	160.07356	160.0736	0.24989	$C_7H_{12}O_4$	142, 115, 71, 59	Pos	2,2-Dimethylglutaric acid
39	10.566	175.06333	175.06248	-4.85539	$C_{10}H_9NO_2$	146, 130, 128, 16, 71, 59	Neg	Indole-3-acetic acid
40	10.629	166.06299	166.06232	-4.03461	$C_9H_{10}O_3$	147, 121, 119, 93, 72	Neg	3-Phenyllactic acid
41	10.714	270.05282	270.0528	-0.07406	$C_{15}H_{10}O_5$	251, 225, 197, 133, 109, 89	Pos	Genistein
42	10.992	164.04734	164.04743	0.54862	$C_9H_8O_3$	145, 119, 91, 72	Neg	3-Coumaric acid
43	11.047	192.04226	192.04245	0.98937	$C_{10}H_8O_4$	178, 165, 133, 105, 91, 79, 67	Pos	Scopoletin
44	11.271	272.06847	272.06844	-0.11027	$C_{15}H_{12}O_5$	153, 147, 123, 119, 91, 68	Pos	Naringenin
45	11.322	145.05276	145.05287	0.75834	C_9H_7NO	147, 146, 118, 91, 65	Pos	4-Indolecarbaldehyde
46	11.373	174.08921	174.0895	1.66581	$C_8H_{14}O_4$	155, 111, 93, 79, 57	Neg	Suberic acid
47	11.466	318.03757	318.03768	0.34587	$C_{15}H_{10}O_8$	301, 291, 273, 263, 245, 195, 165, 153, 109,	Pos	Myricetin
48	11.534	272.06847	272.06844	-0.11027	$C_{15}H_{12}O_5$	255, 153, 147, 119, 91, 68	Pos	Naringenin
49	11.9	464.09548	464.09542	-0.12928	$C_{21}H_{20}O_{12}$	303, 285, 257, 229, 201, 183, 137,	Pos	Hyperoside
50	12.013	272.06847	272.06844	-0.11027	$C_{15}H_{12}O_5$	256, 228, 171, 153, 147, 121, 107,	Pos	Naringenin
51	12.307	138.03169	138.03155	-1.01426	$C_7H_6O_3$	108, 93, 81, 66	Neg	3-Hydroxybenzoic acid
52	12.335	132.07864	132.07842	-1.66567	$C_6H_{12}O_3$	113, 85	Neg	2-Hydroxycaproic acid

53	12.352	448.10056	448.1002	-0.80339	$C_{21}H_{20}O_{11}$	287，269，241，213，161，	Pos	Cynaroside
54	12.497	610.15338	610.15343	0.08195	$C_{27}H_{30}O_{16}$	300，245，151，108，	Neg	Rutin
55	12.523	464.09548	464.09558	0.21547	$C_{21}H_{20}O_{12}$	300，273，245，151，108，	Pos	Quercetin-3β-D-glucoside
56	12.6	248.14124	248.14139	0.60449	$C_{15}H_{20}O_{3}$	231，189，187，161，147，105，93，79，67	Pos	Santamarine
57	12.902	434.08491	434.08448	-0.99059	$C_{20}H_{18}O_{11}$	285,257,229,201,153	Pos	Avicularin
58	12.929	188.10486	188.10424	-3.29603	$C_{9}H_{16}O_{4}$	169，125，97，83，71，57	Neg	Azelaic acid
59	13.051	302.04265	302.0421	-1.82093	$C_{15}H_{10}O_{7}$	257，229，153，137，121，68	Pos	Quercetin
60	13.213	264.13616	264.13551	-2.46085	$C_{15}H_{20}O_{4}$	229，201，187，149，135，121，107，93，83，69	Pos	Ambrosic acid
61	13.527	264.13616	264.13613	-0.11358	$C_{15}H_{20}O_{4}$	247，229，205，173，145，135，117，105，93，83，69	Pos	(±)-Abscisic acid
62	13.875	286.04774	286.04762	-0.41951	$C_{15}H_{10}O_{6}$	259，231，213，185，157，	Pos	Kaempferol
63	13.984	432.10565	432.10575	0.23142	$C_{21}H_{20}O_{10}$	285，255，227，93，65	Neg	Afzelin
64	14.019	316.0583	316.05831	0.03164	$C_{16}H_{12}O_{7}$	274，229，201，153，93，68	Pos	Isorhamnetin
65	14.052	272.06847	272.06883	1.32320	$C_{15}H_{12}O_{5}$	253，187，177，151，119，63	Neg	Naringenin
66	14.129	218.16707	218.16707	0.00000	$C_{15}H_{22}O$	201，159，147，133，105，91，71，55	Pos	Nootkatone
67	14.21	302.04265	302.04255	-0.33108	$C_{15}H_{10}O_{7}$	178，151，121，107，65	Pos	Quercetin
68	14.424	218.16707	218.16707	0.00000	$C_{15}H_{22}O$	301，291，273，263，245，195，165，153，109，	Pos	Zerumbone
69	14.459	202.12051	202.11992	-2.91905	$C_{10}H_{18}O_{4}$	183，139，111，91，69	Neg	3-tert-Butyladipic acid
70	14.927	220.18272	220.18277	0.22708	$C_{15}H_{24}O$	203，161，135，119，107，93，81，71，55	Pos	(-)-Caryophyllene oxide
71	15.267	218.16707	218.16707	0.00000	$C_{15}H_{22}O$	201，159，147，133，105，91，71，55	Pos	Zerumbone
72	15.636	292.20384	292.20381	-0.10267	$C_{18}H_{28}O_{3}$	275，257，239，189，107，93，81，67，55	Pos	9S,13R-12-Oxophytodienoic acid

73	16.161	230.15181	230.15159	-0.95589	$C_{12}H_{22}O_4$	211，167，91，71，57	Neg	Dodecanedioic acid
74	16.198	278.22458	278.22457	-0.03594	$C_{18}H_{30}O_2$	261，243，187，161，147，135，121，109，95，81，67，55	Pos	α-Eleostearic acid
75	17.064	164.08373	164.08394	1.27983	$C_{10}H_{12}O_2$	137，119，105，81，67，55	Pos	4-Phenylbutyric acid
76	17.582	258.18311	258.18321	0.38732	$C_{14}H_{26}O_4$	239，195，	Neg	Tetradecanedioic acid
77	17.754	292.20384	292.2039	0.20534	$C_{18}H_{28}O_3$	273，247，165，93	Pos	12-Oxo phytodienoic acid
78	18.041	278.22458	278.22457	-0.03594	$C_{18}H_{30}O_2$	261，149，121，95，67，57	Pos	α-Linolenic acid
79	18.754	286.21441	286.21446	0.17469	$C_{16}H_{30}O_4$	267，223，195，	Pos	Hexadecanedioic acid
80	19.081	272.23514	272.23538	0.88159	$C_{16}H_{32}O_3$	253，223，151，119，66	Neg	16-Hydroxyhexadecanoic acid
81	19.091	254.22458	254.2245	-0.31468	$C_{16}H_{30}O_2$	237，219，177，149，135，121，109，95，81，69	Pos	Palmitoleic acid
82	19.808	304.24023	304.24019	-0.13148	$C_{20}H_{32}O_2$	259，241，217，149，135，121，107，93，79，67，55	Pos	Arachidonic acid
83	20.04	334.28718	334.2871	-0.23932	$C_{22}H_{38}O_2$	261，243，173，161，133，121，95，81，67	Pos	Docosatrienoic acid
84	20.147	254.22458	254.22451	-0.27535	$C_{16}H_{30}O_2$	237，219，177，153，135，121，109，95，83，69，57	Pos	Palmitoleic acid
85	20.437	178.06299	178.06298	-0.05616	$C_{10}H_{10}O_3$	161，133，119，118，105，93，79，66	Pos	3-Methoxycinnamic acid
86	20.862	299.28243	299.28243	0.00000	$C_{18}H_{37}NO_2$	282，239，62	Pos	Palmitoyl ethanolamide
87	20.89	278.22458	278.22463	0.17971	$C_{18}H_{30}O_2$	259，233，205，91，71，59	Pos	Pinolenic acid
88	21.071	456.36035	456.36042	0.15339	$C_{30}H_{48}O_3$	421，403，367，261，173，145，133，121，109，95，81，	Pos	Oleanolic acid
89	21.146	325.29808	325.29784	-0.73778	$C_{20}H_{39}NO_2$	265，62	Pos	Oleoyl ethanolamide
90	21.174	254.22458	254.22464	0.23601	$C_{16}H_{30}O_2$	209，185，	Neg	Palmitoleic acid
91	21.277	281.27186	281.27193	0.24887	$C_{18}H_{35}NO$	265，247，198，149，114，97，83，69	Pos	Oleamide
92	21.3	456.36035	456.36067	0.70120	$C_{30}H_{48}O_3$	411，277，144，71	Neg	Ursolic acid
93	21.433	272.23514	272.2354	0.95506	$C_{16}H_{32}O_3$	225，197，72	Neg	16-Hydroxyhexadecanoic acid
94	21.525	280.24023	280.24031	0.28547	$C_{18}H_{32}O_2$	261，94，76，59	Neg	Linoleic acid
95	21.83	268.24023	268.24052	1.08112	$C_{17}H_{32}O_2$	214，185，	Neg	trans-10-Heptadecenoic

								acid
96	21.996	283.28751	283.28734	-0.60010	$C_{18}H_{37}NO$	144，116，72	Pos	Stearamide
97	22.238	282.25588	282.25596	0.28343	$C_{18}H_{34}O_2$	90，71	Neg	Oleic acid
98	22.701	328.18859	328.18876	0.51799	$C_{17}H_{28}O_6$	283，265，239，211，24，71	Neg	(-)-Spiculisporic acid
99	23.114	284.27153	284.27177	0.84426	$C_{18}H_{36}O_2$	268，239，	Neg	Stearic acid
100	23.342	337.33447	337.33423	-0.71146	$C_{22}H_{43}NO$	321，303，149，121，97，71，69	Pos	Erucamide
101	24.424	312.30283	312.30308	0.80051	$C_{20}H_{40}O_2$	293，253，183，91	Neg	Arachidic acid
102	26.557	164.08373	164.08394	1.27983	$C_{10}H_{12}O_2$	137，119，105，95，67	Pos	4-Phenylbutyric acid
